# Extensive immune reconstitution inflammatory syndrome in Fingolimod-associated PML: a case report with 7 Tesla MRI data

**DOI:** 10.1186/s12883-019-1407-2

**Published:** 2019-08-09

**Authors:** Tim Sinnecker, Jeffrie Hadisurya, Tilman Schneider-Hohendorf, Nicholas Schwab, Karsten Wrede, Oliver Gembruch, Ralf Gold, Kerstin Hellwig, Sara Pilgram-Pastor, Ortwin Adams, Philipp Albrecht, Hans-Peter Hartung, Orhan Aktas, Markus Kraemer

**Affiliations:** 1 Department of Neurology, Universitätsspital, Basel, Switzerland; 2 Medical Image Analysis Center Basel, Basel, Switzerland; 3 Department of Neurology, Alfried Krupp von Bohlen und Halbach Hospital, Alfried-Krupp-Str. 21, 45117 Essen, Germany; 4 Clinic of Neurology with Institute of Translational Neurology, University Hospital Münster, Münster, Germany; 5 Department of Neurosurgery, University Hospital Essen, University Duisburg-Essen, Essen, Germany; 6 Erwin L. Hahn Institute for Magnetic Resonance Imaging, University Duisburg-Essen, Essen, Germany; 7 Department of Neurology, St. Josef Hospital, Ruhr-University Bochum, Bochum, Germany; 8 Department of Neuroradiology, Alfried Krupp Hospital, Essen, Germany; 9 Institute of Virology, Medical Faculty, Heinrich-Heine University Düsseldorf, Düsseldorf, Germany; 10 Department of Neurology, Medical Faculty, Heinrich-Heine University Düsseldorf, Düsseldorf, Germany

**Keywords:** Progressive multifocal leucencephalopathy, Immune reconstitution inflammatory syndrome, Fingolimod, Multiple sclerosis, 7 tesla MRI

## Abstract

**Background:**

Progressive multifocal leukoencephalopathy (PML) is a rare complication of patients treated with fingolimod.

**Case presentation:**

Routine MRI eventually led to diagnosis of asymptomatic early PML that remained stable after discontinuation of fingolimod. As blood lymphocyte counts normalized, signs of immune reconstitution inflammatory syndrome (IRIS) and renewed MS activity developed. Both, advanced laboratory and ultrahigh field MRI findings elucidated differences between PML and MS.

**Conclusions:**

In our case, early discontinuation of fingolimod yielded a good outcome, lymphocyte counts reflected immune system activity, and paraclinical findings helped to differentiate between PML-IRIS and MS.

## Background

Progressive multifocal leukoencephalopathy (PML) is caused by JC polyomavirus (JCV), and represents a serious adverse complication of effective disease modifying multiple sclerosis (MS) therapies. In addition to natalizumab, PML has been reported during treatment with fingolimod [1]. MRI imaging is crucial to PML diagnosis, but the differentiation between PML, immune reconstitution inflammatory syndrome (IRIS), and renewed MS activity often proves to be very difficult [2].

We here report PML occurrence under sustained fingolimod-associated lymphopenia with development of IRIS after normalization of lymphocytes. Clinical, laboratory and neuroimaging findings including ultrahigh field MRI at 7 Tesla (7 T) are demonstrated to elucidate differentiating features between MS activity, early PML, and IRIS. The patient gave her consent on this publication. The study was conducted according to the principles expressed in the Declaration of Helsinki and 7 T-MRI examination was approved by the University Duisburg-Essen institutional review board.

## Case presentation

A 41-year old woman was diagnosed with relapsing-remitting MS in December 2013. Initial treatment with interferon beta-1b (Extavia®, Betaferon®) was switched to fingolimod in February 2014 due to persistent disease activity. Anti-JCV-serum-antibodies were positive (Unilabs Copenhagen, Denmark). In November 2016 subclinical MRI activity occurred. At this time, the JCV antibody index value was 2.23. Fingolimod was continued, and the absolute lymphocyte count varied between 170/μl and 310/μl.

**Fig. 1:**
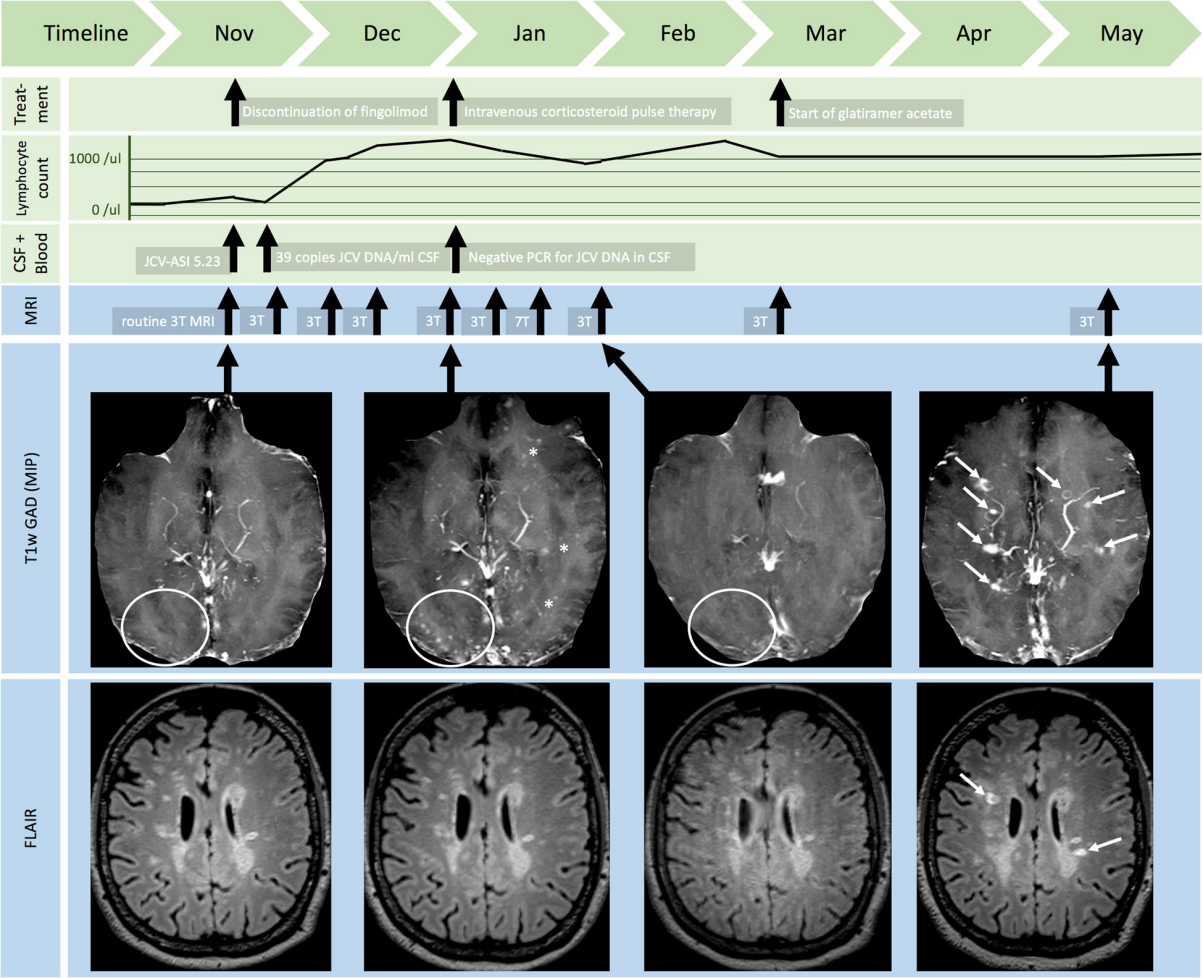
Overview on paraclinical findings and treatment decisions. The figure chronologically displays treatment decisions (first row), lymphocyte counts (second row), laboratory (third row), and MRI findings (fourth and fifth row). At baseline, only very few punctate milky way-like lesions were detectable (circle). After recovery of lymphocytes, new punctate milky way-like lesions developed (circle), and again ceased after corticosteroid pulse therapy (circle). The initial PML lesion in the left hemisphere was stable over time (see Fig. 2). Unfortunately, new MS-like lesions developed after discontinuation of fingolimod (white arrows). Anti-JCV antibody detection and quantification was performed at Unilabs Copenhagen, Denmark. JCV-DNA-PCR from CSF was performed at Institute of Virology, University Clinic of Duesseldorf.

**Fig. 2:**
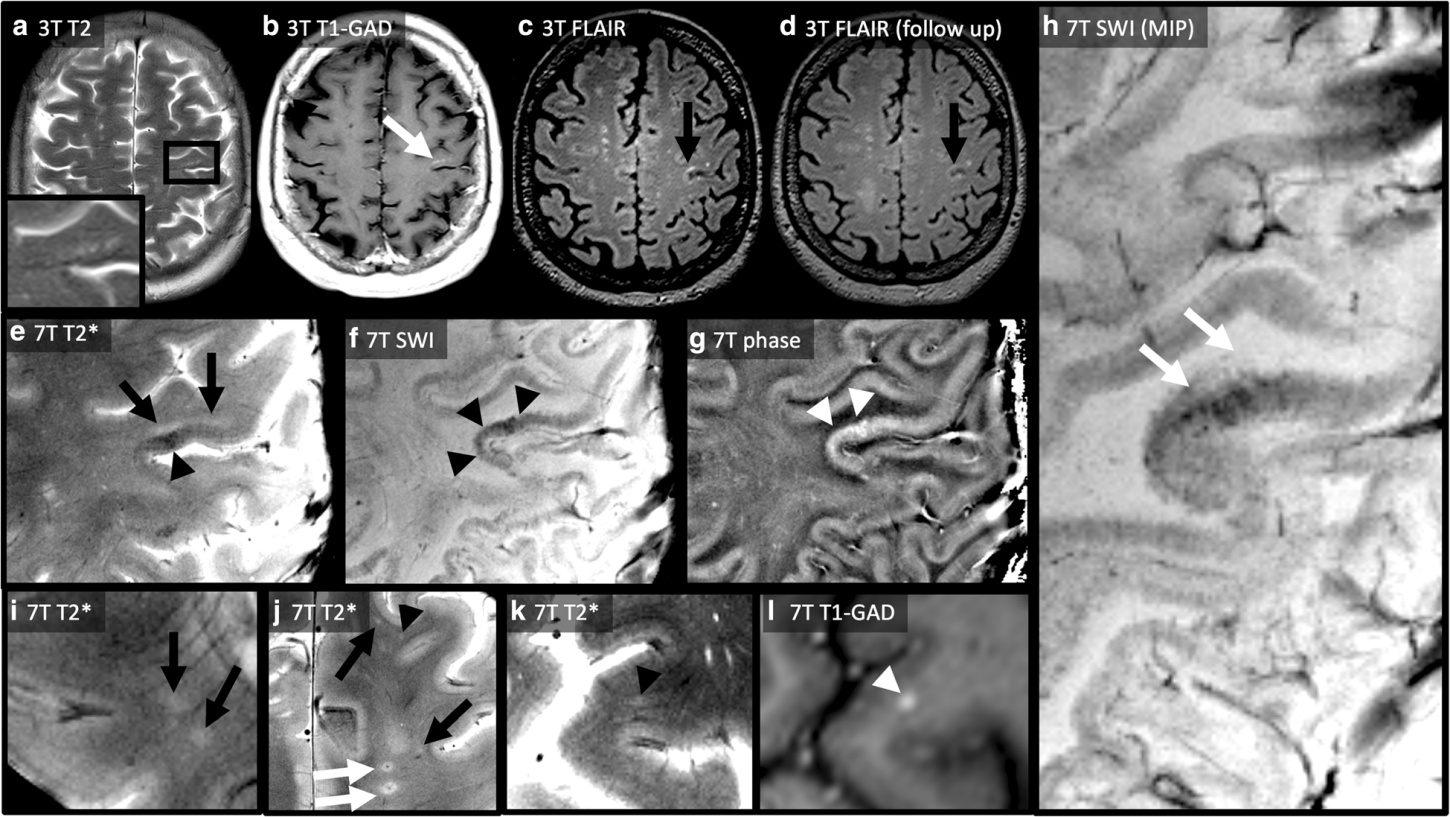
3 T and 7 T MR imaging findings in early fingolimod-associated PML. The first row displays MRI images at baseline (**a**-**c**) and last follow up (**d**). At baseline, T2 weighted imaging (**a**) revealed a new C-shaped lesion with near microcystic appearance (zoom) that clearly infiltrates the short association fibres (“U fibres”). The lesion exhibited a patchy, irregular contrast enhancement (white arrow) on contrast enhanced T1 weighted images (**b**). The lesion was hyperintense on FLAIR (**c**, black arrow). The imaging pattern was strongly suggestive for PML. Thus, fingolimod was stopped immediately. Half year later, the size of the PML lesion remained unchanged (**d**, black arrow). The second and third row shows highly resolving 7 T MRI images. T2*w imaging with a resolution of 0.25 × 0.25mm^2^ delineates a small PML lesion (**e**). The PML lesion is T2*w hyperintense (black arrows), infiltrates the short association fibres (“U fibres”), and appears diffusely delineated against the white matter. Moreover, T2*w hypointense areas are visible within the surrounding cortex (black arrowhead). This finding is more pronounced on susceptibility weighted imaging (SWI, **f**, black arrowheads, “dark” signal). Unwrapped phase maps (**g**) showed positive phase changes (white arrowheads, “bright” signal) indicating paramagnetic effects. A minimal intensity projection map (MIP, **h**) of SWI illustrates SWI hypointense signal along white matter fibre tracts. Moreover, 7 T MRI differentiated between MS- and PML- associated lesions. On the one hand, 7 T T2* weighted MRI visualized a distinct central vein within MS-like lesions (J, white arrows). On top of that, numerous punctate contrast enhancing milky way-like lesions (I-L) were visible. Much of contrast enhancing lesions did not show a central vessel on T2*w images (**i** and **j**, black arrows). A very small vessel was faintly visible in other punctate lesions (**j** and **k**, black arrowheads). **l** demonstrates contrast-enhancement of the lesion displayed in K

One year later, a routine 3 T MRI (14-11-2017) revealed a spotty C-band-shaped hyperintense left-parietal FLAIR lesion with patchy Gadolinium-enhancement, and a microcystic appearance on T2 weighted (T2w) images (Fig. 1 and Fig. 2). Few punctate “milky way”-like Gadolinium-enhancing lesions were present (Fig. 1). Clinically, a slight worsening of the pre-existing gait imbalance and ataxia as well as increased irritability and aggressiveness was noted. The Expanded Disability Status Scale (EDSS) score increased from 4.5 to 5.5.

PML was suspected on the background of atypical lesions on MRI, and subsequently confirmed by detection JCV-DNA in CSF (39 copies/ml, Institute of Virology, Heinrich-Heine-University Duesseldorf; serum JCV antibody index value: 5.23).

**Fig. 3:**
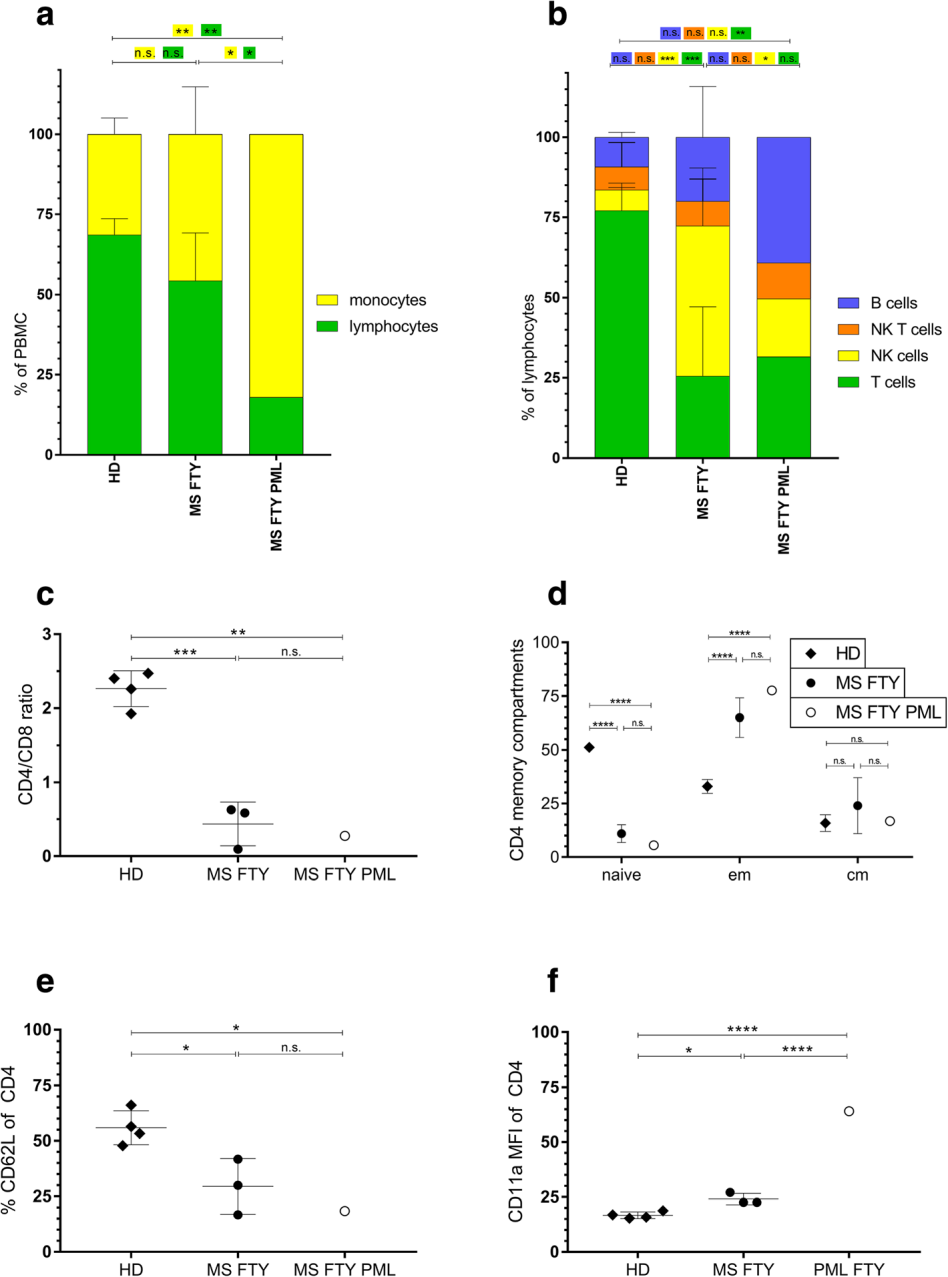
Flow cytometry of peripheral blood-derived mononuclear cells. Peripheral blood mononuclear cells (PBMC) of the patient at the time point of PML diagnosis were analyzed using ten color flow cytometry and compared to four healthy donors (HD) and three Fingolimod-treated control MS patients. (**a**) The proportions of monocytes (CD14+) and lymphocytes (CD3+) among all viable PBMC. (**b**) Proportions of B- (CD19+), NK- (CD56+), NK T- (CD56+, CD3+), as well as T- (CD56-, CD3+) cells of all viable lymphocytes. (**c**) The ratio of CD4+ to CD8+ T cells of all CD3+ T cells. (**d**) Proportions of naïve (CD62L+, CD45RA+), central-memory (CD62L+, CD45RA-) and effector-memory (CD62L-, CD45RA-) CD4+ T cells. (**e**) Percentage of CD62L+ cells of viable CD4+ T cells. (**f**) Mean fluorescence intensity of CD11a on viable CD4+ T cells. Two-way ANOVA with Tukey’s multiple comparisons test was performed for (**a**, **b** and **d**). One-way ANOVA with Tukey’s multiple comparisons test was performed for (**c**, **e** and **f**). * = *p* < 0.05; ** = *p* < 0.01; *** = *p* < 0.001; **** = *p* < 0.0001

Laboratory findings revealed sustained grade 3 lymphopenia (310/μl, CD4+ count 27/μl, CD8+ count 71/μl). Flow cytometry of peripheral blood confirmed a reduction of lymphocytes with reduced proportions of NK cells (CD56+ count 56/μl), as compared to fingolimod-treated control MS patients (Fig. 3a-b). However, the CD4/CD8 ratio and the naïve/memory distribution of T cells was comparable to fingolimod-treated control MS patients (Fig. 3c-d) [3]. The PML biomarker CD62L (L-selectin) was strongly reduced compared to healthy controls and in the lower range of fingolimod-treated MS patients (Fig. 3e) [4]. LFA-1 expression on CD4 T cells was strongly elevated indicating recent cellular activation (Fig. 3f) [5].

Fingolimod was immediately suspended and mirtazapine 30 mg per day was started. Repeated 3 T control MRIs revealed an increasing number of “milky way”-like punctate Gadolinium-enhancing lesions (Fig. 1) in both hemispheres. Concurrently the immune system reconstituted within one month as blood lymphocyte counts normalized (13-12-2018: 1260/μl). Two weeks later, 3 T MRI (27-12-2017) showed substantially more Gadolinium-enhancing punctate lesions (Fig. 1). At this time, JCV-PCR was negative in CSF suggesting IRIS rather than progression of PML, therefore an intravenous corticosteroid pulse therapy was administered (Fig. 1).

The next MRI (10-01-2018) showed slightly less lesions and Gadolinium-enhancement. Clinically, the EDSS score improved to 4.5 (improved gait balance and ataxia).

Ultrahigh field MRI at 7 T was done and visualized the initial C-shaped PML lesion in great anatomical detail (Fig. 2e-h). In addition, T2*w and SWI hypointense (“dark”) areas corresponding to positive (“bright”) MR phase changes and thus indicating paramagnetic susceptibility changes were visible within the surrounding cortex and neighboring white matter fiber tracts (Fig. 2e-h).

Moreover, numerous punctate contrast enhancing milky way-like lesions were detectable on 7 T T1w and T2*w images. Several of these did not present with a central vessel, while a very small vessel was faintly visible in a small proportion of punctate lesions. In contrast, a relatively large central vein was observed within MS-like lesions (Fig. 2i-l).

Follow-up MRIs in March and May 2018 showed new nodular- and ring-like contrast enhancing white matter lesions typical of MS plaques (Fig. 1). Presuming MS activity, immunomodulation with glatiramer acetate was started. The initial left parietal PML lesion did not enlarge at all (Fig. 2d).

## Discussion and conclusions

We here report a case of early fingolimod-associated PML in a patient with MS. The diagnosis was suspected on the background of typical signs of PML in routine MRI and confirmed by positive JCV-DNA-PCR in CSF. After discontinuation of fingolimod, the initial small PML lesion was stable over time and did not enlarge centrifugally into large flame-like PML lesions, which are characteristic for natalizumab-associated PML. Nevertheless, as blood lymphocyte counts normalized and JCV DNA was no longer detectable in CSF, signs of IRIS and renewed MS activity developed. The time interval between PML onset and return of MS disease activity appeared relatively short in comparison to natalizumab-associated PML. Moreover, 7 T MRI revealed distinct imaging patterns and thus helped to differentiate between PML, IRIS and renewed MS activity.

In detail, paramagnetic susceptibility changes as indicated by “dark” signal on SWI/T2*w and “bright” signal on phase maps adjacent to the PML lesion were observed. Their origin is largely unknown, but loss of diamagnetic myelin or iron release by dying oligodendrocytes as an very early sign of JCV infiltration have been discussed.

In addition, we observed numerous “milky way”-like lesions. It has been suggested that these alterations may serve as an early imaging marker for PML [6]. 7 T MRI may add to this finding since a distinct central vein seems to be less frequently detectable within “milky way”-like lesions [7] versus (even small) MS lesions [8].

The origin of “milky way”-like lesions is unknown. One hypothesis is that they highlight an overwhelming immune response, presumably within perivascular spaces. Of note, the absence of a central vessel on highly resolving gradient echo MR images does not contradict the perivascular distribution hypothesis of milky way-like lesions as such sequences do not allow for the visualization of vessels that contain oxygenated blood or are to small for detection (e.g. venules). Other authors have described punctate or milky way–like lesions as areas of active JC virus replication in early PML [7]. Our own observations support both hypotheses as milky-way-like lesions were present from the start (favors the latter), and more milky-way-like lesions developed after normalization of lymphocytes (favors the former). A comparative histopathological and MRI study would have the potential to improve our understanding of milky-way-like lesions. One case report included a histopathological analysis of seven tissue fragments (needle biopsy) in a fingolimod-associated PML patient with milky-way-like lesions on MRI. The study described small inflammatory foci that were in line with a mild host response against JCV infection [9].

Similar to the other recently reported cases [1], our patient presented with lymphopenia (grade 4, in our case) indicating that it may have played a critical role in PML-IRIS development, as suggested from findings on dimethyl fumarate (DMF), where prolonged lymphopenia accounts for most PML cases [10]. However, in contrast to prior hypotheses regarding reduced CD8 cell counts as a potential cause for DMF-PML [11], our case presented with an unaltered CD4/CD8 ratio (in line with the other fingolimod-PML cases [1]), but reduced NK cell numbers.

In summary, in this case PML lesion enlargement stopped after discontinuation of fingolimod in parallel to normalization of lymphocyte counts, and development of signs of IRIS. Our imaging findings support the idea of using ultrahigh field MRI including highly resolving T2*w and SWI to support the diagnosis of PML, and to differentiate from MS activity and IRIS.

## Data Availability

The datasets analyzed during the current study are not publicly available due protection of privacy of the patient.

## Abbreviations

CD8 cells: Cluster of differentition 8 cells
CSF: Cerebral spinal fluid
DMF: Dimethyl fumarate
EDSS: Expanded Disability Status Scale
IRIS: Immune reconstitution inflammatory syndrome
JCV: JC polyomavirus
MRI: Magnetic resonance imaging
MS: Multiple sclerosis
NK cells: Natural killer cells
PML: Progressive multifocal leukoencephalopathy
SWI: Susceptibility-weighted imaging
T: Tesla
T2*w: T2*-weighted imaging
